# Effects of Estradiol on Immunoglobulin G Glycosylation: Mapping of the Downstream Signaling Mechanism

**DOI:** 10.3389/fimmu.2021.680227

**Published:** 2021-05-25

**Authors:** Anika Mijakovac, Julija Jurić, Wendy M. Kohrt, Jasminka Krištić, Domagoj Kifer, Kathleen M. Gavin, Karlo Miškec, Azra Frkatović, Frano Vučković, Marija Pezer, Aleksandar Vojta, Peter A. Nigrović, Vlatka Zoldoš, Gordan Lauc

**Affiliations:** ^1^ Department of Molecular Biology, University of Zagreb Faculty of Science, Zagreb, Croatia; ^2^ Genos Glycoscience Research Laboratory, Zagreb, Croatia; ^3^ Division of Geriatric Medicine, School of Medicine, University of Colorado Anschutz Medical Campus, Aurora, CO, United States; ^4^ Eastern Colorado VA Geriatric Research, Education and Clinical Center, Aurora, CO, United States; ^5^ Faculty of Pharmacy and Biochemistry, University of Zagreb, Zagreb, Croatia; ^6^ Division of Rheumatology, Immunology and Allergy, Brigham and Women´s Hospital, Boston, MA, United States; ^7^ Division of Immunology, Boston Children´s Hospital, Boston, MA, United States

**Keywords:** immunoglobulin G glycosylation, estradiol, CRISPR, inflammation, Runx3

## Abstract

Glycans attached to immunoglobulin G (IgG) directly affect this antibody effector functions and regulate inflammation at several levels. The composition of IgG glycome changes significantly with age. In women, the most notable change coincides with the perimenopausal period. Aiming to investigate the effect of estrogen on IgG glycosylation, we analysed IgG and total serum glycomes in 36 healthy premenopausal women enrolled in a randomized controlled trial of the gonadotropin-releasing hormone analogue (GnRH_AG_) leuprolide acetate to lower gonadal steroids to postmenopausal levels and then randomized to transdermal placebo or estradiol (E_2_) patch. The suppression of gonadal hormones induced significant changes in the IgG glycome, while E_2_ supplementation was sufficient to prevent changes. The observed glycan changes suggest that depletion of E_2_ primarily affects B cell glycosylation, while liver glycosylation stays mostly unchanged. To determine whether previously identified IgG GWAS hits *RUNX1*, *RUNX3*, *SPINK4*, and *ELL2* are involved in downstream signaling mechanisms, linking E_2_ with IgG glycosylation, we used the FreeStyle 293-F transient system expressing IgG antibodies with stably integrated CRISPR/dCas9 expression cassettes for gene up- and downregulation. *RUNX3* and *SPINK4* upregulation using dCas9-VPR resulted in a decreased IgG galactosylation and, in the case of *RUNX3*, a concomitant increase in IgG agalactosylation.

## Introduction

Most proteins in human serum are glycosylated by the covalent addition of diverse glycan structures that fine-tune their function. The regulatory role of glycans has been most extensively explored on immunoglobulin G (IgG) antibodies, where different glycoforms regulate the immune response on multiple levels (1). Glycans attached to the Fc part of the IgG molecule affect interactions with different Fc receptors, which is why changes in glycosylation have direct effects on the immune system at multiple levels (2). IgG glycosylation is altered in many diseases (3), and glycan changes can even appear before the onset of disease symptoms (4–6). In some cases, changes in IgG glycans were shown to be a causative element contributing to the disease development (7–9). IgG glycans that associate with age are known functional effectors of inflammation, and changes in IgG glycosylation seem to be an important factor contributing to ageing at the molecular level (10–12) that can also be used as a biomarker to track individual trajectories of biological ageing (13).

In women, the most prominent change of glycan age coincides with the perimenopausal period (10). A recent intervention study demonstrated that estrogen regulates IgG glycosylation (14), which may explain why perimenopausal females undergo significant changes in the IgG glycome composition. Unfortunately, limitations of the glycoprofiling method used in that study, *i.e.* only IgG galactosylation was estimated from the total plasma glycome profile, prevented us from the detailed characterization of the estrogen effect on IgG glycosylation. In the present study, we aimed at a better understanding of the estrogen role in the regulation of IgG glycosylation, therefore we reanalyzed samples from the previous intervention study (14) using state-of-the-art glycoprofiling technologies (15). We first defined the components of IgG glycome affected by estradiol (E_2_). We then used data from our recent large genome-wide association study (GWAS) of the IgG glycome (16) to identify candidate genes possibly involved in mediating effects of E_2_ on IgG glycosylation. We selected four gene loci, *RUNX1*–*RUNX3*, *SPINK4*, and *ELL2*, involved in E_2_ downstream signaling mechanisms, assuming that these loci represent a part of the molecular pathway linking E_2_ to IgG glycosylation. *In vitro* system used in this study was based on a FreeStyle 293-F (HEK-293FS) transient expression system optimized for secreting a high quantity of native IgG antibodies (16). The system was modified by stable integration of CRISPR/dCas9 expression cassette containing either VPR (for gene upregulation) or KRAB (for gene downregulation). Using this system, we were able to demonstrate the effects of selected genes on specific IgG glycans which were previously associated with biological ageing.

## Methods

### Institutional Approval

This study was conducted at the University of Colorado Anschutz Medical Campus (CU-AMC). All procedures were performed in accordance with the ethical standards and approved by the Colorado Multiple Institutional Review Board (COMIRB) and the Scientific Advisory and Review Committee at the University of Colorado Anschutz Medical Campus (CU-AMC). The study was registered on ClinicalTrials.gov (NCT00687739) on May 28, 2008.

### Participants and Screening Procedures

Participants were healthy eumenorrheic premenopausal women who volunteered to take part in the study. All volunteers underwent screening procedures, as described previously (17). The main inclusion criteria were age (25 to 49 years) and regular menstrual cycle function [no missed cycles in the previous year, cycle length 28 ± 5 days and confirmation of ovulatory status (ClearPlan Easy, Unipath Diagnostics, Waltham, MA)]. Exclusion criteria were pregnancy or lactation, hormonal contraception, oral glucocorticoids or diabetes medications, smoking, and body mass index (BMI) >39 kg/m^2^. Following the Declaration of Helsinki, all volunteers provided written informed consent to participate, with the knowledge that the risks of the study included menopause-like effects (e.g., weight gain, bone loss, menopausal symptoms).

### Experimental Design and Study Procedures

The parental trial was a randomized, double-blinded, placebo-controlled trial to determine the effects of estradiol (E_2_) deficiency on body composition, bone mineral density, components of energy expenditure and physical activity in premenopausal women (17, 18). In short, all participants underwent suppression of ovarian sex hormones with gonadotropin-releasing hormone agonist therapy (GnRH_AG_, leuprolide acetate 3.75 mg, Lupron; TAP Pharmaceutical Products, Inc; Lake Forest, IL) in the form of monthly intramuscular injections. A single injection of leuprolide acetate produces an initial stimulation (for 1 to 3 weeks) followed by a prolonged suppression of pituitary gonadotropins FSH and LH, while repeated monthly dosing suppresses ovarian hormone secretion (19). A urine pregnancy test confirmed the absence of pregnancy before each dosing. After completing the screening procedures, eligible volunteers underwent baseline testing during the early follicular phase (days 2 to 6 after the onset of menses) of the menstrual cycle. At the beginning of the following menstrual cycle, participants began 5-months of GnRH_AG_ therapy to suppress ovarian function. Participants were randomized to receive either transdermal E_2_ 0.075 mg/d (Bayer HealthCare Pharmaceuticals, Berkeley, CA) or placebo patches (GnRH_AG_ + E_2_, n = 15; GnRH_AG_ + PL, n = 21). The E_2_ regimen kept serum E_2_ concentrations in the mid-to-late follicular phase range (100 to 150 pg/ml). To reduce the risk of endometrial hyperplasia and minimize exposure to progesterone, women randomized to E_2_ received medroxyprogesterone acetate (5 mg/d, as a pill) for 12 days every other month (end of months 2 and 4, and after completion of follow-up testing). During these monthly visits, participants were under supervision of the research nurse practitioner. Participants were asked to report health and medication use changes (e.g., doctor visits, hospitalizations), as well as any study-related problems/concerns over the past 4 weeks.

### Sample Collection

Blood samples were collected at three timepoints: during baseline testing (T1), during week 20 of the hormonal intervention (T2), and at the spontaneous recovery of the normal menstrual cycle function, approximately 4-months after completion of the drug intervention (T3). A single sample (~5 ml) was obtained in the morning (~8 AM), after an overnight fast (at least 10 h). Baseline samples were obtained immediately before the first GnRH_AG_ injection. Serum was separated from each collected sample upon blood withdrawal and stored at −80°C until analysis.

### Sex Hormone Concentration

Collected sera were analyzed for numerous sex hormones. Estrone (E1), estradiol (E2) and progesterone (P) concentrations were determined by radioimmunoassay (RIA, Diagnostic Systems Lab, Webster, TX). Total testosterone (T) concentration was determined by chemiluminescence immunoassay (Beckman Coulter, Inc. Fullerton, CA), and sex hormone-binding globulin (SHBG) concentration was determined by immunoradiometric assay (Diagnostic Systems Laboratory).

### Isolation of Immunoglobulin G, Release and Labeling of N-Glycans From IgG

The whole procedure was performed according to the already published protocol (20). In short, IgG was isolated from sera (100 μl) by affinity chromatography using a 96-well plate with protein G coupled to a monolithic stationary phase (BIA Separations, Slovenia). The isolated IgG was denatured with the addition of SDS (Invitrogen, USA) and incubation at 65°C, after which the excess of SDS was neutralized with Igepal CA-630 (Sigma-Aldrich, USA). N-glycans were released from IgG with the addition of PNGase F (Promega, USA) in a PBS buffer during the overnight incubation at 37°C. The released glycans were fluorescently labelled with 2-AB dye (Merck, Germany) in the 2 h incubation at 65°C. Free label and reducing agent were removed from the samples by hydrophilic interaction liquid chromatography solid phase extraction (HILIC-SPE). IgG N-glycans were eluted with ultrapure water and stored at −20°C.

### Release and Labeling of N-Glycans From Total Serum Proteins

The whole procedure was performed as described previously (4). In short, serum proteins (10 μl) were denatured by SDS and incubated at 65°C. Excess SDS was neutralized by Igepal CA-630 (Sigma-Aldrich, USA). Serum proteins were deglycosylated by PNGase F (Promega, USA) in a PBS buffer during the overnight incubation at 37°C. Released glycans were fluorescently labelled with 2-AB dye (Merck, Germany) in the 2h incubation time at 65°C. Excess of reagents and proteins from previous steps was removed by hydrophilic interaction liquid chromatography solid phase extraction (HILIC-SPE). Serum N-glycans were eluted with ultra-pure water and stored at −20°C.

### Hydrophilic Interaction Chromatography (HILIC)-UPLC Analysis of Labeled Glycans

Fluorescently labeled N-glycans were separated by ultra-performance liquid chromatography (UPLC) on a Waters Acquity UPLC *H*-Class Instrument consisting of a sample manager, quaternary solvent manager, and a fluorescence (FLR) detector set with excitation and emission wavelengths at 250 and 428 nm, respectively. The UPLC system was under the control of Empower 3 software, build 3471 (Waters, USA). Labeled N-glycans were separated on an amide ACQUITY UPLC^®^ Glycan BEH chromatography column (Waters, USA), 100 × 2.1 mm i.d. for IgG glycans and 150 × 2.1 mm for glycans from total serum proteins, 1.7 μm BEH particles, with 100 mM ammonium formate pH 4.4 as solvent A, and 100% acetonitrile as solvent B. The separation method used a linear gradient of 75–62% acetonitrile at a flow rate of 0.40 ml/min in a 27 min analytical run for IgG glycans and a linear gradient of 70–53% acetonitrile at a flow rate of 0.561 ml/min in a 23 min analytical run for glycans from total serum proteins. Samples were kept at 10°C before injection onto the column. The separation temperature of the column was 60°C for the IgG glycans and 25°C for glycans from serum proteins. Data processing included an automatic integration method that was manually corrected to maintain the same intervals of chromatographic integration across all samples. Chromatograms were separated in the same manner into 24 peaks for IgG N-glycans and 39 peaks for N-glycans from total serum proteins. The abundance of glycans in each chromatographic peak was expressed as a percentage of the total integrated area (% area).

### Plasmid Constructs

Plasmid constructs pORF-hp21 and pORF-hp27 used to enhance protein production were obtained from Invivogen, while p3SVLT was constructed by cloning a codon-optimized version of the SV40 large T antigen coding region (16) in pcDNA3 (Addgene). Unwanted BsaI restriction sites in IgG heavy and light chain (kindly provided by Gestur Vidarsson, Sanquin, Amsterdam) were removed using QuikChange Lightning Site-Directed Mutagenesis Kit (Agilent). IgG chains were then cloned into pUK21gg for the subsequent Golden Gate cloning step. Expression plasmids encoding gene-specific guide RNA (gRNA) molecules were constructed in the multi-guide system described by Josipović et al. (21). Three gRNA molecules were cloned individually in a backbone plasmid pSgMx-A or pSgMx-G (where x represents the order of gRNA molecules; 1,2,3 and A or G represents Cas9 ortholog (dSaCas9 or dSpCas9 respectively) it recognizes) (22) for each gene: *RUNX1, RUNX3, SPINK4* and *ELL2.* gRNA molecules for *RUNX1, RUNX3*, *ELL2* and *SPINK4* were then cloned in pSgx3 as modular “multiguide” molecules. Two non-targeting gRNA molecules and one gRNA molecule targeting *B4GALT1* for dSaCas9 and dSpCas9 were cloned in the same way described above. Together with modules for antibody heavy chain (HC) and light chain (LC), Cbh promoter and bGH terminator, single guide or multiguide RNA molecules were cloned in a backbone pBackBone-BZ by modular Golden Gate cloning method described in Josipović et al. (21). Sequences of gRNA molecules and details of plasmids/modules used in Golden Gate cloning are given in **Supplementary Tables 6, 7**.

### Cell Culture and Transfections

Stable cell lines PB-dSaCas9-VPR-1 and PB-dSpCas9-KRAB-3 were established from FreeStyle™ 293-F cells (Gibco) with piggyBac transposon system by limiting dilution method (unpublished data) and maintained in FreeStyle™ 293 Expression Medium (Gibco) in 125 ml Erlenmeyer flasks (Nalgene) and cultivated at 37°C in the atmosphere with 8% CO_2_ on PSU-20i Multi-functional Orbital Shaker at 140 rpm according to the protocol from Vink et al. (16). Transfections of stable cell lines were done using 293fectin Transfection Reagent (Gibco) according to the manufacturer’s protocol optimized for 2 ml per well. When cells reached ≥90 viability, they were plated in non-treated 6-well plates at a concentration of 500,000 cells/ml and were transfected with 2 μg of plasmids diluted in Opti-MEM I Reduced Serum Medium (Gibco) to reach a volume of 80 ml. For enhanced expression of immunoglobulin G, cells were transfected with a plasmid containing gRNA and IgG heavy and light chain, p3SVLT, pORF-hp21 and pORF-hp27 in the ratio: 0.69/0.01/0.05/0.25 (16). Cells were collected 5 days after transfection by centrifugation at 4,000*g* (5 min). The cell pellet was used for gene expression profiling, while the supernatant was used for glycan analysis.

### Quantitative Real-Time PCR (qPCR)

For gene expression profiling, total RNA was extracted with RNeasy Mini Kit (Qiagen) from cell pellets collected five days after transfection. Reverse transcription was done on 50 ng of total isolated RNA using the PrimeScript RTase (TaKaRa) and random hexamer primers (Invitrogen) for TaqMan Gene Expression Assay or on 5 ng of total isolated RNA (pretreated with TURBO DNase (Invitrogen)) for SYBR Green Gene Expression Assay. Both variants of RT-qPCR were performed according to the manufacturer’s protocol using the 7500 Fast Real-Time PCR System using TaqMan Gene Expression Master Mix with the following assays: Hs01021970_m1 (RUNX1), Hs00205508_m1 (SPINK4), Hs01023022_m1 (ELL2), Hs00155245_m1 (B4GALT1) and Hs02800695_m1 (HPRT1) or PowerUp SYBR Green Master Mix with primer sequences listed in **Supplementary Table 8**. The mean value of 12 replicates was normalized to the expression of the *HPRT1* gene as endogenous control and was analysed using the ΔΔCt method (23). Fold change (FC) was shown relative to gene expression in cells transfected with a plasmid expressing non-targeting gRNA.

### IgG Isolation From FreeStyle™ 293-F Cells, N-Glycan Release, Labeling and HILIC-UPLC Analysis

IgG was isolated from FreeStyle 293-F cell culture supernatants using Protein G Agarose fast flow beads (Merck, Germany). The beads were prewashed three times with 10× bead volume of 1× PBS. In each washing step, beads were resuspended in 1× PBS, centrifugated at 150×*g* for 10 s, and the supernatant was removed. After the last wash, prewashed beads were resuspended in 1× PBS to make a 50:50 (v/v) beads slurry. Approximately 2 ml of FreeStyle 293-F cell culture supernatant were mixed with an equal volume of 1× PBS and 40 µl of prepared 50% bead slurry in a 5 ml tube. The samples were resuspended by pipetting action and incubated 1 h at room temperature with gentle shaking to allow IgG to bind to the beads. During the incubation period, the samples were resuspended twice by pipetting action. After incubation, the samples were centrifugated at 150×*g* for 10 s, and the supernatants were then carefully removed and discarded. The beads were washed three times with 300 µl of 1× PBS and three times with 300 µl of ultrapure water to remove non*-*specifically bound proteins. After washing steps, bound IgG was eluted by incubating the beads in 100 µl of 0.1 M formic acid (Merck) for 15 min at room temperature with gentle shaking. Eluted IgG was neutralised with 17 µl of 1M ammonium bicarbonate (Merck, Germany*).* IgG concentration in the eluate was measured using Nanodrop 8000 (Thermo Scientific, USA). Samples were subsequently dried in a vacuum concentrator.

N-glycan release, glycan labeling, clean-up of glycans and separation of glycans by HILIC-UHPLC were performed according to a previously established protocol (20) with some modifications. Briefly, dried IgG was denatured by SDS (Invitrogen, USA) and heated at 65°C. The excess of SDS was neutralised with Igepal CA-630 (Merck, Germany), and N-glycans were released by 18 h of incubation with PNGaseF (Promega, USA). The released glycans were fluorescently labeled with procainamide in a two-step reaction. In the first step, 25 µl of freshly prepared labeling solution, containing 172.8 mg/ml of procainamide hydrochloride (Thermo Fisher Scientific, USA) in a mixture of DMSO (Merck, Germany) and glacial acetic acid (Merck, Germany) (70:30, v/v), was added to each sample followed by incubation for 1 h at 65°C. Then in the next step, 25 µl of freshly prepared solution, containing 179.2 mg/ml of 2-picoline borane as a reducing agent in a mixture of DMSO and acetic acid (70:30, v/v), was added to each sample followed by incubation for 1.5 h at 65°C. Free label and reducing agent were removed from the samples using hydrophilic interaction liquid chromatography solid-phase extraction (HILIC-SPE) on a 0.2 μm GHP filter plate (Pall Corporation, USA). Glycans were eluted with ultrapure water and stored at −20°C until use. Fluorescently labeled N-glycans were separated by hydrophilic interaction chromatography on a Waters Acquity UPLC instrument (Waters, USA) consisting of a quaternary solvent manager, sample manager and FLR fluorescence detector set with excitation and emission wavelengths of 310 and 370 nm, respectively. The instrument was under the control of Empower 3 software, build 3471 (Waters, USA). Labeled N-glycans were separated on a Waters BEH Glycan chromatography column, 100 × 2.1 mm i.d., 1.7 μm BEH particles, with 100 mM ammonium formate, pH 4.4, as solvent A and ACN as solvent B. The separation method used a linear gradient of 75–62% ACN (v/v) at a flow rate of 0.4 ml/min over 31 min. Samples were maintained at 10°C before injection, and the separation temperature was 60°C. The system was calibrated using an external standard of hydrolyzed and procainamide-labeled glucose oligomers from which the retention times for the individual glycans were converted to glucose units (GU). Data processing was performed using an automatic processing method with a traditional integration algorithm, after which each chromatogram was manually corrected to maintain the same intervals of integration for all the samples. The chromatograms were separated in the same manner as chromatograms of human plasma/serum-derived IgG glycans into 24 peaks, and the abundance of glycans in each peak was expressed as a percentage of the total integrated area. The structural assignment of the glycans present in the chromatographic peaks was done based on i) overlay with the chromatogram of human plasma IgG glycans for which structures corresponding to each peak had been previously determined (24) and ii) GU values of the glycan peaks using the GlycoStore database (www.glycostore.org).

### Statistical Analysis

The area under chromatogram peaks was normalized to total chromatogram area, then each glycan peak was logit transformed, and batch corrected using ComBat method (R package ‘sva’) (25). Data were back transformed, and derived glycan traits were calculated as a sum or ratio of selected directly measured glycan peaks based on particular glycosylation features (i.e. sialylation or fucosylation).

Mixed models were used to estimate the effect of the intervention (R package ‘lme4’) (26). Hormone concentration or particular glycan level was set as dependent variable and timepoint (with levels: *baseline*, *after intervention* and *after recovery*) nested within the treatment group (*placebo* and *estradiol*) as independent variables. Also, the model was age-adjusted, and the subject’s ID was included as a random intercept to account for variation between the subjects.

Change in estradiol and change in glycan abundance were calculated by subtracting values of consecutive timepoints. Mixed models were used to estimate the relationship between the change in glycans and the change in estradiol concentration. mixed models were used. The change in glycan abundance was defined as a dependent variable, while the change in estradiol concentration was defined as a fixed effect. Group and time period nested within the group were defined as random factors. Both change in glycan abundance and change in estradiol concentration were transformed to a standard normal distribution by inverse transformation of ranks to normality.

Prior modeling, glycan levels were transformed to a standard normal distribution by inverse transformation of ranks to normality (R package ‘GenABEL’) (27), while hormone concentrations were log transformed. Based on fitted models, changes of dependent variables after intervention or recovery (relative to baseline) were compared between the groups (placebo vs estradiol) using *post-hoc* t-test. False discovery rate was controlled using the Benjamini–Hochberg method at a significance level of 0.05.

Differences between groups for gene expression and glycan levels following CRISPR/dCas9 manipulations were tested using the non-parametric Mann–Whitney test. Results with p <0.05 were considered statistically significant. All statistical analyses were performed in R programming software (version 3.6.3) (28).

## Results

### IgG Glycome Is Affected by Estradiol

**Figure 1:**
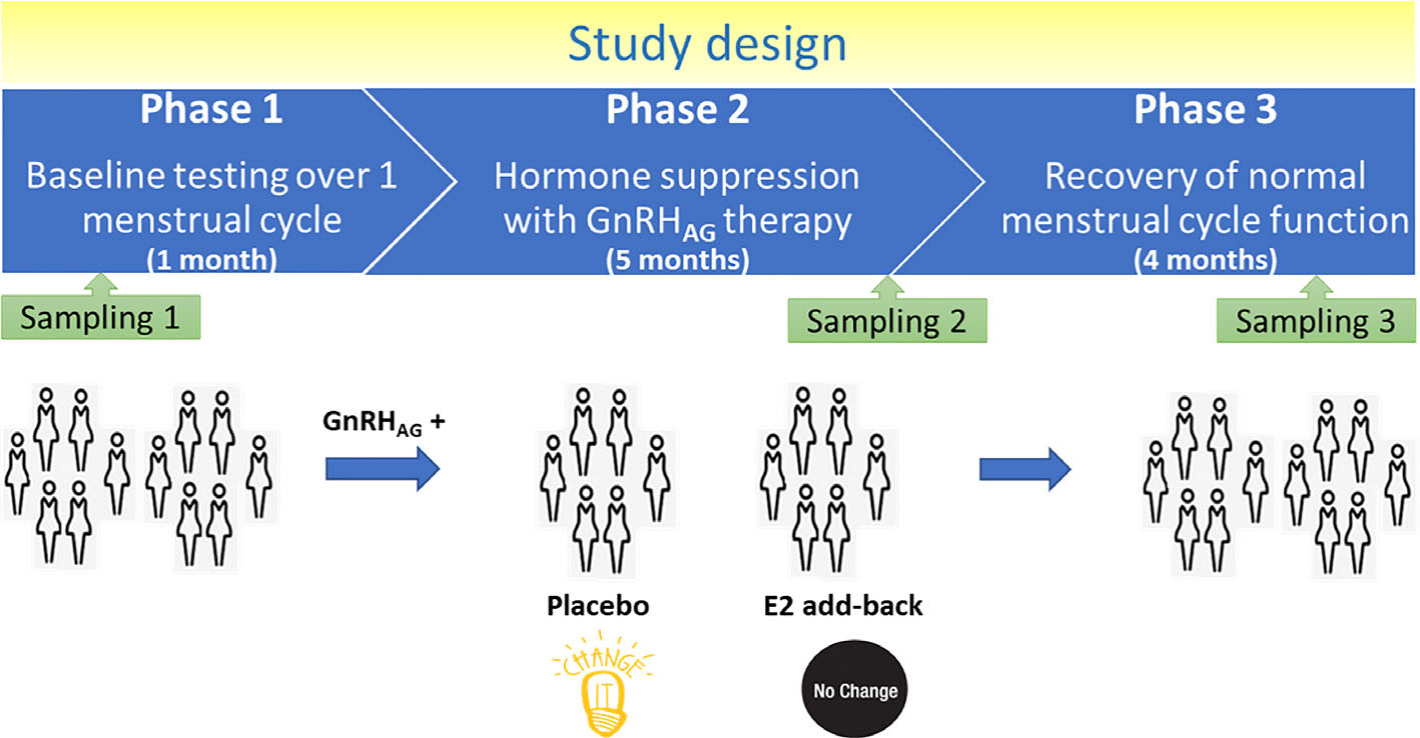
Design of the study.

**Figure 2:**
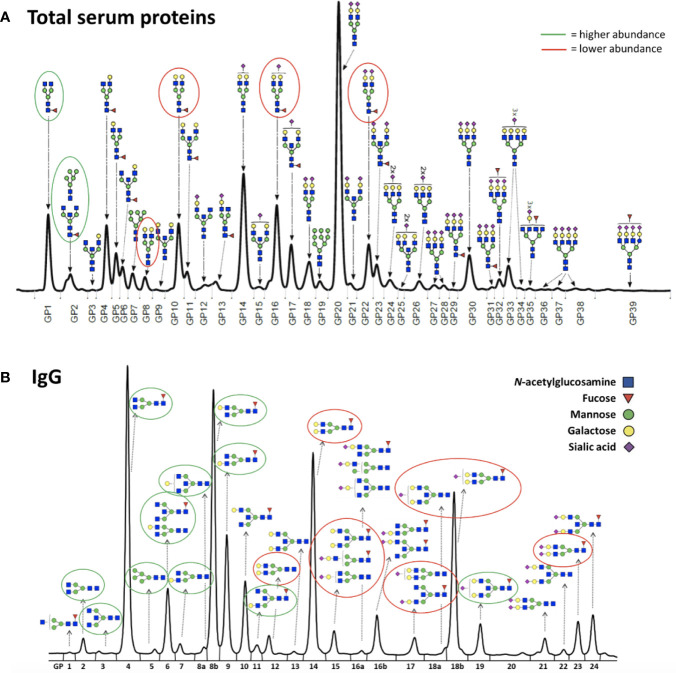
Representative chromatograms of **(A)** the total serum glycome and **(B)** the IgG glycome. Glycans that decreased after the gonadal hormone suppression with gonadotropin-releasing hormone analogue leuprolide acetate (GnRH_AG_) therapy are circled in red, and those that increased are circled in green.

**Figure 3:**
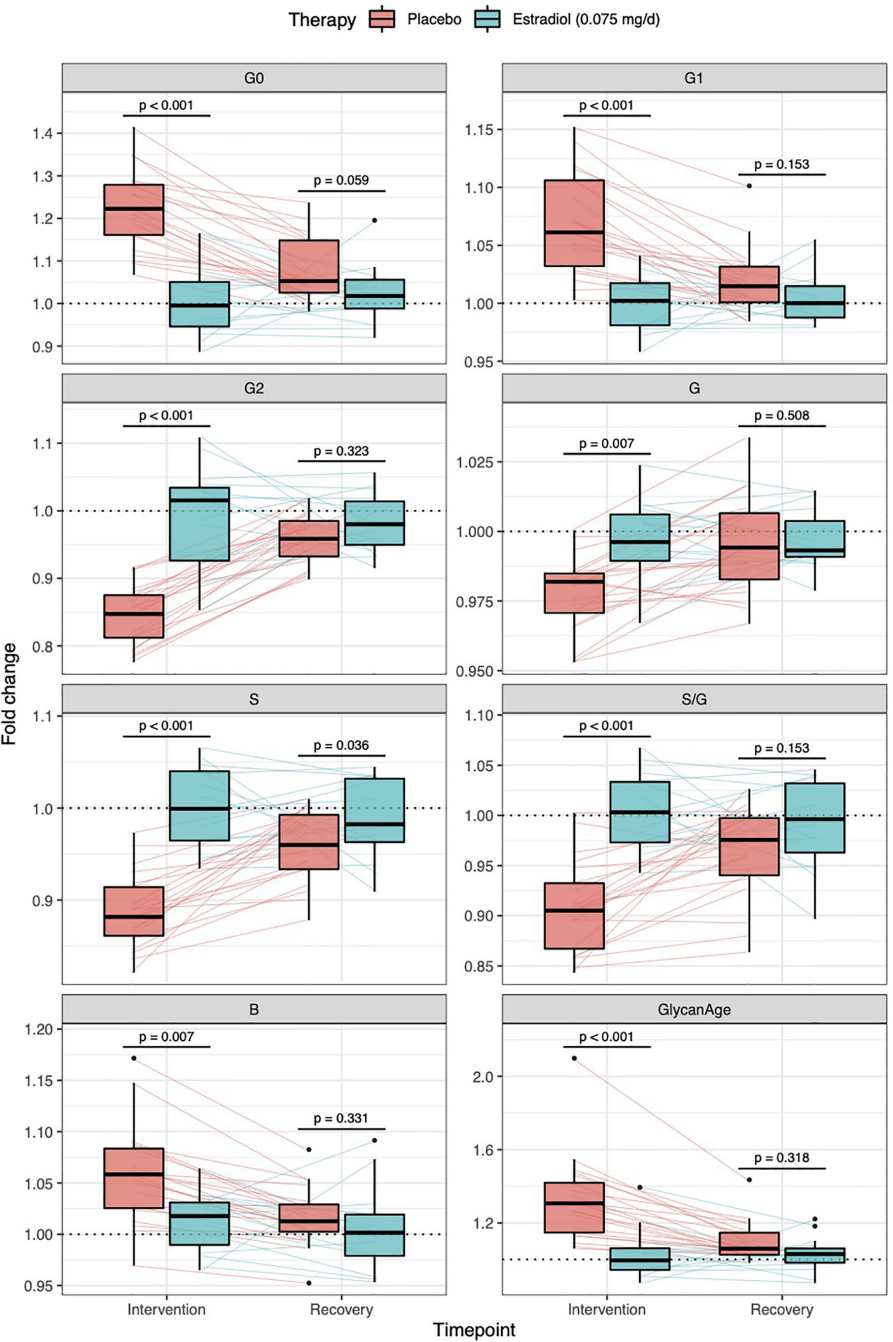
Effects of gonadal hormone suppression on IgG glycosylation. Gonadotropin-releasing hormone analogue leuprolide acetate (GnRH_AG_) was used to lower gonadal steroids to postmenopausal levels in healthy premenopausal women (n = 36) that were then randomized to transdermal placebo (n = 21) or estradiol patch (n = 15). Changes in the IgG glycome composition after five months of GnRH_AG_ (Intervention) with supplementation of E_2_ (transdermal estradiol supplementation) or without supplementation of E_2_ (supplementation with placebo) and four months after the end of the intervention (Recovery) are shown on the graph. G2, digalactosylated glycans; G1, monogalactosylated glycans; G0, agalactosylated glycans; S, sialylated glycans; S/G, ratio of sialylation and galactosylation; B, glycans with bisecting GlcNAc; G, all glycans with galactose.

Thirty-six healthy premenopausal women were enrolled in a randomized controlled trial of the gonadotropin-releasing hormone analogue (GnRH_AG_) leuprolide acetate to lower gonadal steroids to postmenopausal levels and then randomized to transdermal placebo (PL) or estradiol (E_2_) patch (**Figure 1**) (17). In order to analyse total serum and IgG glycomes, serum samples were collected: at baseline (Sampling 1); after five months of GnRH_AG_ administration with concurrent supplementation with either E_2_ or placebo (Sampling 2); and four months after the end of the intervention, when natural hormonal cycling was restored (Sampling 3). **Figure 2** shows representative UHPLC chromatograms of the total serum (**Figure 2A**) and IgG (**Figure 2B**) glycomes and the direction of glycan changes after the suppression of gonadal hormones. IgG glycosylation analysis revealed significant changes in IgG glycome composition after gonadal hormone suppression (time point at the end of Phase 2), while E_2_ supplementation was sufficient to prevent changes in the IgG glycome composition (**Figure 3**, **Table 1** and **Supplementary Table 1**). After four months of the recovery period (Sampling 3 after the end of Phase 3), the IgG glycome composition returned to nearly pre-intervention values in the placebo group. Galactosylation was the most affected IgG glycome feature, with a significant decrease of digalactosylated glycans (G2) and an increase of monogalactosylated (G1) and agalactosylated (G0) glycans. The level of sialylated glycans (S) and the ratio of sialylation and galactosylation (S/G) of IgG significantly decreased, while the abundance of glycans with bisecting GlcNAc (B) increased. Only the abundance of core-fucosylated (F) glycans did not change by depletion of E_2_. IgG glycosylation traits related to galactosylation and sialylation, which were particularly affected by the suppression of E_2,_ are also the main components of the glycan age clock of the biological age. This index has initially been developed to predict chronological age (10) but was subsequently converted into the test of biological age (29). Suppression of E_2_ resulted in a median increase of GlycanAge by 9.1 years, which was completely attenuated by E_2_ add-back (**Supplementary Figure 1**). At the individual level, the extent of changes in hormone concentration (**Supplementary Table 2**) correlated moderately with the extent of changes in individual IgG glycans (**Supplementary Table 3**), suggesting that other factors (beside gonadal hormones) also strongly affect the composition of the IgG glycome.

**Table 1:** Glycan abundances (%) of directly measured IgG glycan traits at the baseline and deviations from the baseline after intervention and after recovery timepoint.

Glycan	Intervention	Glycan Abundance (%) at Baseline median (IQR)	Difference in glycan abundance (%) relative to baseline. sampling after:			
			Intervention	p_I_	Recovery	p_R_
			median (IQR)		median (IQR)	
**GP1**	Placebo	0.049	0.010	1.95 × 10^−1^	0.002	1.48 × 10^−1^
		(0.040–0.051)	(0.008–0.012)		(0.000–0.010)	
	Estradiol	0.069	0.009		−0.001	
		(0.041–0.091)	(0.000–0.010)		(−0.007–0.006)	
**GP2**	Placebo	0.269	0.102	**3.53 × 10** ^−^ **^6^**	0.027	1.48 × 10^−1^
		(0.199–0.654)	(0.082–0.148)		(−0.010–0.042)	
	Estradiol	0.361	−0.003		0.005	
		(0.261–0.495)	(−0.047–0.031)		(−0.026–0.028)	
**GP3**	Placebo	0.059	0.011	**4.08 × 10** ^−^ **^4^**	0.003	7.56 × 10^−2^
		(0.059–0.070)	(0.009–0.013)		(−0.001–0.011)	
	Estradiol	0.070	−0.006		−0.001	
		(0.061–0.096)	(−0.010–0.004)		(−0.008–0.003)	
**GP4**	Placebo	14.0	3.08	**2.46 × 10** ^−^ **^8^**	0.869	6.58 × 10^−2^
		(12.4–17.6)	(2.35–3.93)		(0.345–1.771)	
	Estradiol	17.3	−0.152		0.187	
		(14.0–19.2)	(−1.003–1.031)		(−0.374–0.847)	
**GP5**	Placebo	0.158	0.021	**2.56 × 10** ^−^ **^4^**	0.009	**3.22 × 10** ^−^ **^2^**
		(0.143–0.180)	(0.011–0.028)		(−0.004–0.015)	
	Estradiol	0.162	−0.004		−0.004	
		(0.155–0.194)	(−0.010–0.006)		(−0.013–0.005)	
**GP6**	Placebo	3.44	0.673	**1.17 × 10** ^−^ **^7^**	0.200	1.48 × 10^−1^
		(2.98–4.28)	(0.567–0.824)		(0.124–0.301)	
	Estradiol	3.71	0.067		0.126	
		(3.28–4.36)	(−0.114–0.253)		(−0.112–0.198)	
**GP7**	Placebo	0.363	0.039	**3.51 × 10** ^−^ **^3^**	0.010	4.56 × 10^−1^
		(0.240–0.466)	(0.020–0.056)		(−0.013–0.026)	
	Estradiol	0.394	−0.010		0.000	
		(0.289–0.435)	(−0.029–0.019)		(−0.016–0.014)	
**GP8**	Placebo	18.5	0.990	**2.22 × 10** ^−^ **^5^**	0.139	4.91 × 10^−1^
		(17.5–19.9)	(0.604–1.246)		(−0.189–0.474)	
	Estradiol	20.0	−0.030		0.032	
		(18.5–21.0)	(−0.351–0.208)		(−0.316–0.207)	
**GP9**	Placebo	9.66	0.843	**1.14 × 10** ^−^ **^7^**	0.137	3.64 × 10^−1^
		(8.31–10.73)	(0.560–1.274)		(−0.015–0.405)	
	Estradiol	9.69	−0.024		0.069	
		(9.09–10.31)	(−0.285–0.221)		(−0.050–0.313)	
**GP10**	Placebo	4.99	0.177	5.28 × 10^−1^	0.087	3.50 × 10^−1^
		(4.69–5.38)	(0.127–0.358)		(0.029–0.175)	
	Estradiol	4.65	0.125		−0.040	
		(4.37–5.52)	(0.020–0.167)		(−0.104–0.047)	
**GP11**	Placebo	0.636	0.051	**2.46 × 10** ^−^ **^4^**	0.018	5.77 × 10^−1^
		(0.587–0.696)	(0.030–0.092)		(0.000–0.031)	
	Estradiol	0.665	0.000		0.015	
		(0.555–0.715)	(−0.020–0.036)		(−0.004–0.030)	
**GP12**	Placebo	1.010	−0.178	**1.19 × 10** ^−^ **^5^**	−0.021	9.52 × 10^−1^
		(0.621–1.333)	(−0.256 to −0.129)		(−0.060–0.000)	
	Estradiol	0.781	0.000		−0.020	
		(0.596–0.984)	(−0.095–0.020)		(−0.062–0.010)	
**GP13**	Placebo	0.249	−0.010	2.57 × 10^−1^	−0.009	4.90 × 10^−1^
		(0.211–0.281)	(−0.029–0.011)		(−0.019–0.010)	
	Estradiol	0.230	0.008		0.001	
		(0.210–0.245)	(−0.020–0.019)		(−0.015–0.011)	
**GP14**	Placebo	20.1	−2.95	**4.71 × 10** ^−^ **^7^**	−0.735	3.12 × 10^−1^
		(16.6–21.9)	(−3.93 to −2.11)		(−1.383 to −0.315)	
	Estradiol	16.3	0.212		−0.469	
		(14.8–18.8)	(−0.987–0.708)		(−0.977–0.253)	
**GP15**	Placebo	1.98	−0.250	**5.39 × 10** ^−^ **^7^**	−0.031	3.68 × 10^−1^
		(1.76–2.39)	(−0.337 to −0.129)		(−0.107–0.018)	
	Estradiol	1.90	0.002		−0.041	
		(1.55–2.05)	(−0.040–0.075)		(−0.059–0.053)	
**GP16**	Placebo	3.09	0.010	4.78 × 10^−1^	−0.019	7.14 × 10^−2^
		(2.54–3.38)	(−0.059–0.122)		(−0.088–0.039)	
	Estradiol	3.05	0.041		0.026	
		(2.89–3.26)	(−0.010–0.073)		(−0.002–0.067)	
**GP17**	Placebo	1.043	−0.100	**2.75 × 10** ^−^ **^5^**	−0.034	1.95 × 10^−1^
		(0.835–1.086)	(−0.177 to −0.060)		(−0.060 to −0.011)	
	Estradiol	0.895	−0.010		0.008	
		(0.761–0.990)	(−0.037–0.031)		(−0.048–0.016)	
**GP18**	Placebo	12.9	−2.42	**9.92 × 10** ^−^ **^9^**	−0.585	3.23 × 10^−1^
		(10.3–14.1)	(−2.99 to −1.92)		(−1.129 to −0.156)	
	Estradiol	11.0	−0.009		−0.383	
		(10.1–12.3)	(−0.717–0.823)		(−0.513–0.248)	
**GP19**	Placebo	1.87	0.062	**9.19 × 10** ^−^ **^3^**	−0.043	9.72 × 10^−1^
		(1.72–1.99)	(0.000–0.089)		(−0.064–0.039)	
	Estradiol	1.88	0.000		−0.001	
		(1.61–2.18)	(−0.034–0.050)		(−0.065–0.017)	
**GP20**	Placebo	0.418	−0.010	**1.75 × 10** ^−^ **^2^**	−0.014	3.68 × 10^−1^
		(0.381–0.443)	(−0.041–0.010)		(−0.048–0.007)	
	Estradiol	0.389	0.010		0.006	
		(0.346–0.418)	(−0.010–0.048)		(−0.014–0.019)	
**GP21**	Placebo	0.851	−0.029	3.98 × 10^−1^	−0.007	7.58 × 10^−1^
		(0.762–0.974)	(−0.051–0.022)		(−0.062–0.073)	
	Estradiol	0.790	0.010		0.000	
		(0.725–0.815)	(−0.032–0.036)		(−0.034–0.027)	
**GP22**	Placebo	0.12	0.001	2.81 × 10^−1^	−0.010	8.83 × 10^−1^
		(0.11–0.16)	(−0.020–0.010)		(−0.010–0.001)	
	Estradiol	0.120	0.001		0.001	
		(0.111–0.135)	(0.000–0.010)		(−0.010–0.010)	
**GP23**	Placebo	1.90	−0.178	**5.17 × 10** ^−^ **^3^**	−0.034	1.48 × 10^−1^
		(1.70–2.09)	(−0.218 to −0.100)		(−0.157–0.028)	
	Estradiol	1.91	−0.032		0.028	
		(1.57–2.21)	(−0.115–0.014)		(−0.112–0.061)	
**GP24**	Placebo	1.89	0.050	1.53 × 10^−1^	−0.060	6.09 × 10^−1^
		(1.65–2.10)	(−0.059–0.108)		(−0.089–0.027)	
	Estradiol	1.92	−0.008		−0.020	
		(1.62–2.22)	(−0.035–0.045)		(−0.068–0.028)	

p values describe statistical significance of difference between estradiol and placebo group after intervention (p_I_) and recovery (p_R_). p values smaller than 0.05 are bolded. IQR, limits of the interquartile range (1st–3rd quartile); GP, glycan peak.

**Figure 4:**
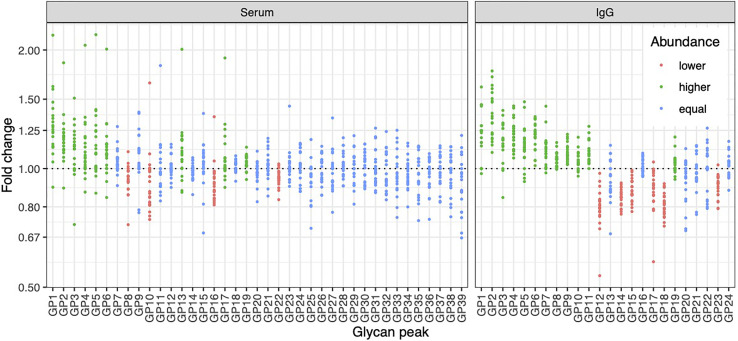
Effects of gonadal hormone depletion on total plasma glycans and IgG glycans. Gonadotropin-releasing hormone analogue leuprolide acetate (GnRH_AG_) was used to lower gonadal steroids to postmenopausal levels in healthy premenopausal women (n = 36) that were then randomized to transdermal placebo (n = 21) or estradiol patch (n = 15). Changes in the total plasma glycome and IgG glycome composition after five months of GnRH_AG_ without supplementation of E_2_ (supplementation with placebo) are shown. Each dot is a change in a single individual. Changes that are statistically significant after correction for multiple testing are shown in red (statistically significant decrease) or in green (statistically significant increase).

To determine whether the effects of E_2_ on glycosylation were restricted to IgG, we also analysed total serum protein N-glycome in the same samples. Changes observed in the total serum N-glycome (**Figure 4**) were restricted only to some neutral glycans and core-fucosylated sialylated biantennary glycans known to originate nearly exclusively from immunoglobulins (30). This suggests that depletion of E_2_ affects B cell (IgG) glycosylation, while liver glycosylation does not seem to be affected, at least not in a way that would alter proportions of individual non-immunoglobulin *N*-glycans in the total serum glycome (**Figure 2A**).

### Downstream Signaling Mechanisms Linking Estradiol With IgG Glycan Traits

Glycans are inherited as complex traits defined by multiple genes (31, 32) which play a role in the synthesis and variation of individual glycan structures. Through a series of GWAS papers in the last decade (32–35), we mapped an extensive network of genes that potentially regulate the glycosylation of IgG. Using the Signalling Pathways Project (SPP) web knowledgebase (36), we explored the effects of E_2_ on GWAS hits for IgG galactosylation and sialylation (35). Results presented in **Supplementary Figure 2** indicate that E_2_ affects the expression of B4GALT1, glycosyltransferase which adds galactose to IgG glycans, but also the genes which are not directly involved in IgG glycosylation, such as the *RUNX1*–*RUNX3* loci (Runt-related transcription factor 1 and RUNX family transcription factor 3, found in many promoters and enhancers, which can either activate or suppress transcription) and the *SPINK4* locus (serine peptidase inhibitor, also known as PEC-60). These genes were identified as GWAS hits for IgG galactosylation. In addition, *ELL2* (Elongation Factor for RNA Polymerase 2), another GWAS hit for IgG glycosylation, more specifically sialylation, also appears to be strongly regulated by E_2_. On the other hand, there were no conclusive results on ST6GAL1, the enzyme that adds sialic acid to IgG.

**Figure 5:**
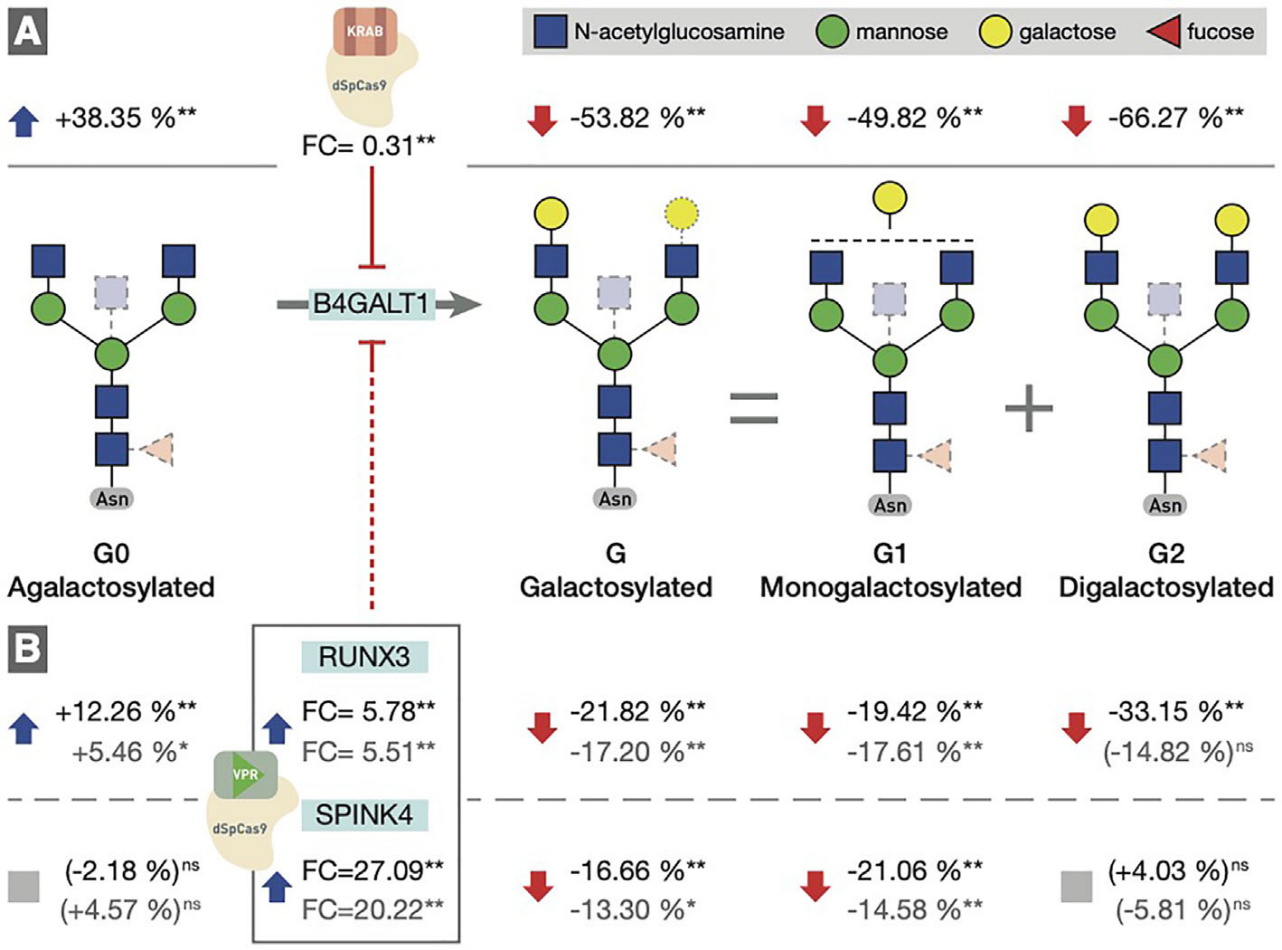
**(A)** Downregulation of the *B4GALT1* gene by dCas9-KRAB induced changes in IgG galactosylation. Fold change (FC) between cells in which *B4GALT1* was directly downregulated by dCas9/KRAB and control cells (containing non-targeting gRNA) was 0.31, and subsequent change was recorded in IgG glycan phenotype: an increase of agalactosylated glycan structures appeared with a concomitant decrease in mono- and digalactosylated glycans (G1, G2). Corresponding changes in glycan structures are given as a relative change with non-targeting gRNA glycan levels as a baseline. Agalactosylated glycan structures (G0) are converted to galactosylated structures (G) by the enzymatic activity of B4GALT1. **(B)** Changes in IgG glycosylation resulting from upregulation of RUNX3 and SPINK4 by dCas9/VPR. FC values between cells in which *RUNX3* and *SPINK4* were directly upregulated by dCas9/VPR and control cells (containing non-targeting gRNA) are given for the first experiment (indicated in black) and the replicate (indicated in gray). The resulting putative inhibition of *B4GALT1* was confirmed indirectly by the effect on the glycosylation profile. The indirect and speculative nature of RUNX3/SPINK4 effect on galactosylation is indicated by the dashed red line. Statistical significance: *<0.05; **<0.01; ns, not significant.

To determine the involvement of the *RUNX1*–*RUNX3*, *SPINK4*, and *ELL2* loci in downstream signaling mechanisms linking E_2_ with IgG glycosylation, we directly manipulated their transcriptional activity in the *in vitro* IgG expression system HEK-293FS using CRISPR/dCas9 molecular tools and subsequently analysed IgG glycan phenotype. A recently developed HEK-293FS transient system for IgG secretion with stably integrated CRISPR/dCas9 expression cassette for gene upregulation (dCas9-VPR) and downregulation (dCas9-KRAB) was used for this purpose. These cells were transfected with a plasmid containing genes for IgG heavy and light chains aiming to induce the production and secretion of IgG antibodies. Described plasmid also contains specifically designed gRNAs targeting the appropriate fusion constructs (either dCas9-VPR or dCas9-KRAB) to candidate genes *RUNX1*, *RUNX3, SPINK4*, and *ELL2.* As a proof of concept (*i.e.*, positive control), we targeted dCas9-KRAB to the promoter region of the *B4GALT1* gene, coding for a glycosyltransferase responsible for IgG galactosylation. We observed a significant decrease in the *B4GALT1* gene expression level and subsequent decrease in galactosylated glycans, with a concomitant increase in agalactosylated glycans, as expected (**Figure 5A**). Subsequently, we upregulated *RUNX1*, *RUNX3*, and *SPINK4* genes using dCas9-VPR and downregulated *RUNX1*, *RUNX3* and *ELL2* genes using dCas9-KRAB. Using specific gRNA for our targets, we found significant changes in the expression of *RUNX1*/VPR, *RUNX3*/VPR, *RUNX3*/KRAB, and *SPINK4*/VPR. The changes in gene expression were replicated in two independent sets of experiments (**Supplementary Table 4**). However, only the changes in *RUNX3*/VPR and *SPINK4*/VPR were accompanied by significant change in IgG glycosylation profile, in both cases related to the level of IgG galactosylation (**Figure 5B**). We found a decrease in galactosylated glycans upon upregulation of *RUNX3* and *SPINK4* and a concomitant increase in agalactosylated glycans in the case of *RUNX3* (**Figure 5B**). The dataset for all glycan traits is available in **Supplementary Table 5**.

## Discussion

The composition of IgG glycome is an essential aspect in the regulation of the immune system (1). However, molecular mechanisms contributing to changes of the IgG glycome composition are only vaguely understood. Here we show that estradiol is an important factor in regulating IgG glycosylation in women and that its effects on N-glycosylation are limited explicitly to B cells, as depletion of E_2_ did not cause N-glycosylation changes of other serum proteins. Previous analysis of the same cohort of patients demonstrated that depletion of E_2_ decreases galactosylation of IgG (14), and here we expand this finding to the decrease of sialylation and an increase of bisecting GlcNAc. Particularly interesting is the change in the ratio of sialylation and galactosylation (S/G, **Figure 3**, **Supplementary Table 1**), suggesting that the depletion of gonadal hormones directly affects sialylation and that the decrease in sialylation is not just a reflection of decreased galactose levels (needed for subsequent sialylation). It was previously reported that estrogen affects the expression of the *ST6GAL1* gene in both mice and humans (37). Unfortunately, we were not able to prove this using ours *in vitro* transient expression system. Therefore mechanistic aspects of this association remain to be demonstrated.

When mechanisms regulating protein glycosylation are investigated, the focus is always on the expression of glycosyltransferases, the enzymes that synthesize glycans (38). Nevertheless, in general, there is a slight correlation between glycosyltransferase expression levels and levels of glycans it synthesizes (39), which indicates that regulatory mechanisms may be more complex than a simple change in the expression of glycosyltransferases. Indeed, in a series of GWAS studies performed in the last decade, we identified a network of at least 30 genes associated with and potentially involved in regulating IgG glycosylation (32–35).

One of the potential mechanisms by which E_2_ could increase IgG galactosylation is through direct activation of the B4GALT1 galactosyltransferase, which adds galactose to IgG. *In vitro* studies showed that both overexpression of estrogen receptor (40) and treatment of cells with E_2_ leads to increased expression of the *B4GALT1* gene (41, 42). Our study decreased *B4GALT1* expression using CRISPR/dCas9-KRAB fusion in a unique IgG-secreting model cell system resulting in the expected change of the IgG glycome composition. It confirmed the importance of this critical biosynthetic enzyme for IgG galactosylation and proved the efficacy of our FreeStyle 293-F cell system, containing stably integrated dCas9-VPR and -KRAB and secreting IgG, for functional validation of GWAS hits for IgG glycosylation.

By analysing the Signalling Pathways Project (SPP) web knowledgebase (36), we found that several other GWAS hits for the IgG glycosylation, which are not glycosyltransferases but genes with other functions, such as transcription factors *RUNX1* and *RUNX3*, as well as *SPINK4* and *ELL2*, also seem to be regulated by estradiol. However, the fact that these genes were both involved in the regulation of IgG glycosylation and affected by E_2_ is not strong enough to prove their direct involvement in B cell IgG glycosylation because their effects could also be through an indirect mechanism. Using CRISPR/dCas9 molecular tools, we were able to increase the expression of *RUNX3*, which resulted in lower levels of IgG galactosylation (**Figure 5**).

With this experiment, we confirmed that *RUNX3* is involved in regulating IgG glycosylation in our *in vitro* model system of B cells (16). The first experimental validation of this GWAS hit positions *RUNX3* as a potential target for pharmacological interventions. *RUNX3* gene downregulation may improve IgG galactosylation and sialylation and may have potential anti-inflammatory effects. Activation of *SPINK4* had similar effects on the IgG glycome composition as activation of *RUNX3*. However, because the basal expression of *SPINK4* in HEK-293FS cells was low, we could not confirm if its suppression would have the opposite effect. Furthermore, *SPINK4* is located in relative proximity to *B4GALT1* (i.e., 50 kb distance). Therefore, although we did not observe a statistically significant change in transcript levels at the time of analysis, we cannot exclude the possibility that the binding of the dCas9-VPR fusion construct, used for the activation of SPINK4 in the region between *SPINK4* and *B4GALT1*, negatively affected *B4GALT1* expression. One putative mechanism could be spurious upregulation of *B4GALT1* antisense RNA 1 (*B4GALT1*-AS1), which is also located in this region, although we have not verified this hypothesis experimentally. We did not observe any statistically significant effects of *ELL2* on IgG glycosylation. This was not surprising because *ELL2* is a GWAS hit for sialylation and our *in vitro* expression system produces IgG antibodies with very low levels of sialic acid which presents a difficulty for evaluation of effects on sialylation. Therefore, the role of *ELL2* in the regulation of IgG glycosylation by estrogen still needs further exploration.

For the first time, the molecular mechanism through which E2 could regulate IgG glycosylation has been identified and functionally validated in the present study. Considering multiple functional roles of IgG glycans in balancing the immune system, this pathway may be a target for the future development of a new class of anti-inflammatory drugs acting downstream of E_2_ and having only a subset of the molecular consequences of hormone therapy.

## Data Availability Statement

The raw data supporting the conclusions of this article will be made available by the authors, without undue reservation.

## Ethics Statement

The studies involving human participants were reviewed and approved by the Scientific Advisory and Review Committee at the University of Colorado Anschutz Medical Campus. The patients/participants provided their written informed consent to participate in this study.

## Author Contributions

GL, VZ, and PN designed the study. JJ, JK, and MP performed glycosylation analysis and interpreted glycan data. AM and KM performed CRISPR/dCas9 gene manipulations in HEK-293FS cells, collected IgG antibodies, and interpreted the data. WK and KG performed the intervention study. DK, AF, FV and AV analysed the data. GL wrote the initial draft of the manuscript. All authors contributed to the article and approved the submitted version.

## Funding

Glycosylation analysis was performed in Genos Glycoscience Research Laboratory and partly supported by the European Union’s Horizon 2020 grant IMForFuture (grant #721815), European Structural and Investment Funds grants “Centre of Competence in Molecular Diagnostics grant” (#KK.01.2.2.03.0006), and “Croatian National Centre of Research Excellence in Personalized Healthcare” (#KK.01.1.1.01.0010) and European Regional Development Fund, under grant agreement No. KK.01.1.1.04.0085, project “Genomic engineering and gene regulation in cell lines and model organisms by CRISPR/Cas9 technology—CasMouse”.

## Conflict of Interest

GL is the founder and owner of Genos Ltd, a private research organization that specializes in high-throughput glycomic analyses and has several patents in this field. JJ, JK, AF, FV and MP are employees of Genos Ltd.

The remaining authors declare that the research was conducted in the absence of any commercial or financial relationships that could be construed as a potential conflict of interest.

The handling editor declared a past co-authorship with one of the authors GL.
