# Supplementary material for: Effects of Estradiol on Immunoglobulin G Glycosylation: Mapping of the Downstream Signaling Mechanism
Source: Front Immunol. 2021 May 25;12:680227. doi: 10.3389/fimmu.2021.680227 (PMC8186398; doi:10.3389/fimmu.2021.680227)
Supplement: Supplementary file 1 [file DataSheet_1.pdf]

Effects of estradiol on immunoglobulin G glycosylation: mapping of the downstream signalling mechanism

Anika Mijakovac[1,*], Julija Jurić[2,*], Wendy M. Kohrt[3,4], Jasminka Krištić[2], Domagoj Kifer[5], Kathleen M. Gavin[3,4], Karlo Miškec[1], Azra Frkatović[2], Frano Vučković[2], Marija Pezer[2], Aleksandar Vojta[1], Peter A. Nigrović[6,7], Vlatka Zoldoš[1,a], Gordan Lauc[2,5,a]

[1] Department of Molecular Biology, University of Zagreb Faculty of Science, Zagreb, Croatia
[2] Genos Glycoscience Research Laboratory, Zagreb, Croatia
[3] Division of Geriatric Medicine, School of Medicine, University of Colorado Anschutz Medical Campus, Aurora, Colorado, USA
[4] Eastern Colorado VA Geriatric Research, Education and Clinical Center, Aurora, Colorado, USA
[5] Faculty of Pharmacy and Biochemistry, University of Zagreb, Zagreb, Croatia
[6] Division of Rheumatology, Immunology and Allergy, Brigham and Women´s Hospital, Boston, Massachusetts, USA
[7] Division of Immunology, Boston Children´s Hospital, Boston, Massachusetts, USA

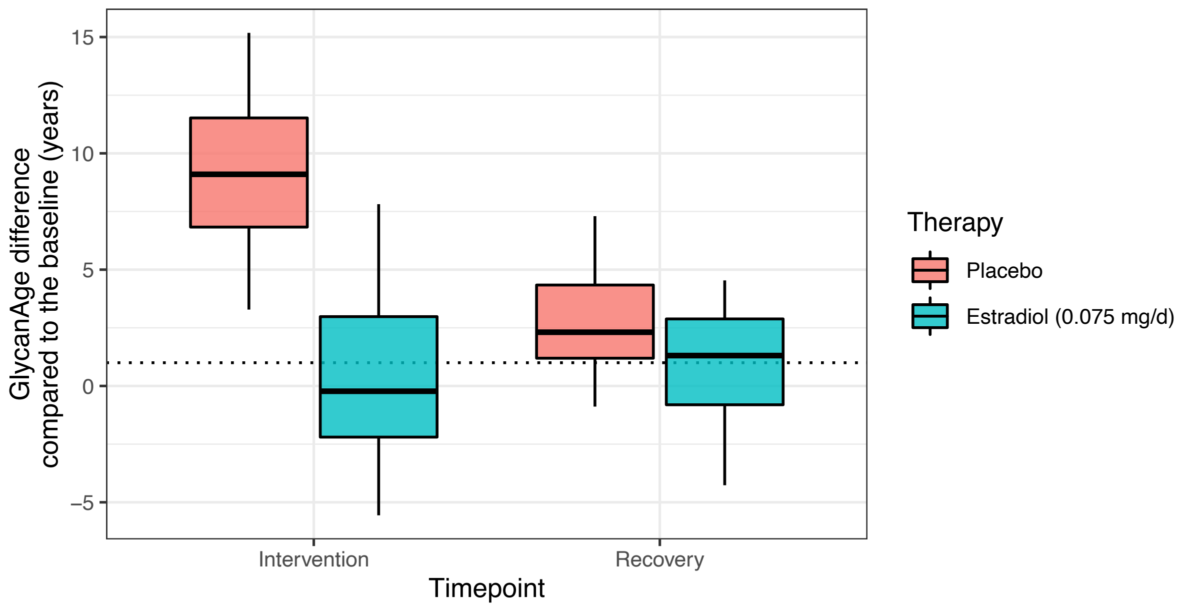

Supplementary Figure 1. Effects of gonadal hormone suppression on GlycanAge. Gonadotropin-releasing hormone analogue leuprolide acetate ($GnRH_{AG}$) was used to lower gonadal steroids to postmenopausal levels in healthy premenopausal women (n=36) that were then randomized to transdermal placebo (n=21) or estradiol patch (n=15). Changes in the GlycanAge after five months of $GnRH_{AG}$ (Intervention) with supplementation of E_2 (transdermal estradiol supplementation) or without supplementation of E_2 (supplementation with placebo), and four months after the end of the intervention (Recovery) are shown on the graph.

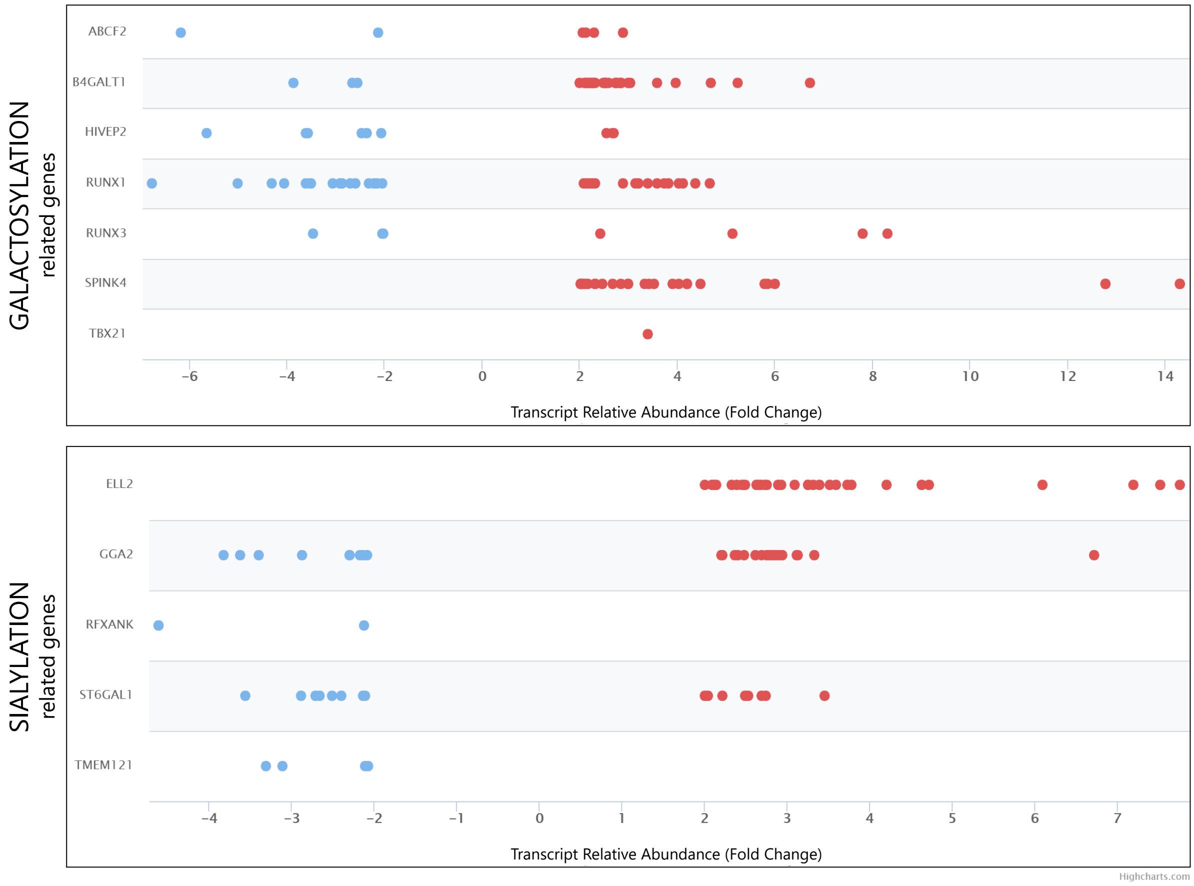

Supplementary Figure 2. Effects of estradiol E_2 on expression of genes, GWAS hits for IgG galactosylation and sialylation.

Supplementary Table 1. Glycan abundances (%) of derived glycan traits at the baseline and deviations from the baseline after intervention and after recovery timepoint.

**GLYCAN**	**INTERVENTION**	**GLYCAN ABUNDANCE (%) AT BASELINE** MEDIAN (IQR)	**DIFFERENCE IN GLYCAN ABUNDANCE (%) RELATIVE TO BASELINE. SAMPLING AFTER:**			
			**Intervention** median (IQR)	p_I	**Recovery** median (IQR)	p_R
**B**	Placebo	15.9 (14.8 - 16.8)	0.889 (0.445 - 1.026)	$\mathbf{7,10\times10^{-3}}$	0.168 (0.036 - 0.492)	$3,31\times10^{-1}$
	Estradiol	15.9 (14.2 - 16.6)	0.281 (-0.155 - 0.469)		0.027 (-0.290 - 0.284)	
**CF**	Placebo	95.4 (94.8 - 96.3)	0.118 (-0.095 - 0.311)	$2,35\times10^{-1}$	0.052 (-0.029 - 0.145)	$7,70\times10^{-1}$
	Estradiol	95.8 (95.4 - 96.4)	-0.026 (-0.124 - 0.087)		0.027 (-0.134 - 0.159)	
**HM**	Placebo	0.158 (0.143 - 0.180)	0.021 (0.011 - 0.028)	$\mathbf{2,56\times10^{-4}}$	0.009 (-0.004 - 0.015)	$\mathbf{3,22\times10^{-2}}$
	Estradiol	0.162 (0.155 - 0.194)	-0.004 (-0.010 - 0.006)		-0.004 (-0.013 - 0.005)	
**G0**	Placebo	17.3 (16.0 - 21.3)	3.99 (3.18 - 4.70)	$\mathbf{9,10\times10^{-9}}$	1.118 (0.541 - 1.992)	$5,86\times10^{-2}$
	Estradiol	21.5 (17.8 - 24.1)	-0.156 (-1.171 - 1.351)		0.321 (-0.341 - 1.132)	
**G1**	Placebo	34.1 (33.0 - 35.7)	1.98 (1.21 - 3.35)	$\mathbf{4,38\times10^{-7}}$	0.510 (0.032 - 1.044)	$1,53\times10^{-1}$
	Estradiol	35.5 (34.8 - 36.4)	0.070 (-0.678 - 0.633)		0.004 (-0.424 - 0.522)	
**G2**	Placebo	23.9 (19.8 - 25.6)	-3.37 (-4.51 - -2.52)	$\mathbf{1,14\times10^{-7}}$	-0.744 (-1.610 - - 0.299)	$3,23\times10^{-1}$
	Estradiol	19.0 (17.2 - 22.3)	0.282 (-1.181 - 0.743)		-0.321 (-1.032 - 0.215)	
**S0**	Placebo	76.3 (73.8 - 78.0)	2.62 (1.96 - 3.30)	$\mathbf{2,69\times10^{-9}}$	0.787 (0.211 - 1.512)	$6,40\times10^{-2}$
	Estradiol	77.9 (75.9 - 78.8)	0.008 (-1.022 - 0.759)		0.323 (-0.325 - 0.745)	
**S1**	Placebo	18.3 (16.6 - 20.0)	-2.53 (-3.28 - -2.01)	$\mathbf{7,96\times10^{-9}}$	-0.654 (-1.491 - - 0.273)	$1,58\times10^{-1}$
	Estradiol	16.9 (16.2 - 18.7)	0.157 (-0.650 - 0.910)		-0.330 (-0.576 - 0.400)	
**S2**	Placebo	4.84 (4.34 - 5.17)	-0.127 (-0.328 - - 0.043)	$9,43\times10^{-2}$	-0.073 (-0.336 - 0.029)	$1,58\times10^{-1}$
	Estradiol	4.57 (4.20 - 5.22)	-0.034 (-0.085 - 0.030)		-0.023 (-0.204 - 0.077)	
**G**	Placebo	57.0 (55.3 - 59.1)	-1.062 (-1.777 - - 0.825)	$\mathbf{6,64\times10^{-3}}$	-0.331 (-0.981 - 0.389)	$5,08\times10^{-1}$
	Estradiol	54.8 (53.1 - 58.5)	-0.221 (-0.600 - 0.315)		-0.391 (-0.526 - 0.228)	

**S**	Placebo	23.1 (21.3 - 25.3)	-2.56 (-3.34 - -2.22)	**$4{,}00\times10^{-9}$**	-0.800 (-1.525 - -0.153)	**$3{,}64\times10^{-2}$**
	Estradiol	21.7 (20.4 - 23.5)	-0.020 (-0.719 - 0.937)		-0.353 (-0.786 - 0.609)	
**S/G**	Placebo	40.4 (37.5 - 44.0)	-3.79 (-5.50 - -2.78)	**$1{,}88\times10^{-6}$**	-1.169 (-2.206 - -0.095)	$1{,}53\times10^{-1}$
	Estradiol	37.8 (36.4 - 44.1)	0.125 (-0.987 - 1.377)		-0.179 (-1.353 - 1.054)	

p values describe statistical significance of difference between estradiol and placebo group after intervention (p_I) and recovery (p_R). p values smaller than 0.05 are bolded. IQR – limits of the interquartile range (1st quartile – 3rd quartile). B – bisecting N-acetylglucosamine; CF – core fucosylation, HM – high mannose; G0 – agalactosylated; G1 – monogalactosylated; G2 – digalactosylated; S0 – asialylated; S1 – monosialylated, S2 - disialylated structures. G = G1 + G2, S = S1 + S2, S/G = S / G.

Supplementary Table 2. Concentration of hormones at the baseline and differences from the baseline after intervention and after recovery timepoint.

VARIABLE	INTERVENTION	CONCENTRATION AT BASELINE median (IQR)	DIFFERENCE IN CONCENTRATION RELATIVE TO BASELINE. SAMPLING AFTER: Intervention median (IQR)	p_I	Recovery median (IQR)	p_R
**estradiol** *pg/mL*	Placebo	54.0 (44.5 - 79.2)	-31.5 (-53.5 - -19.8)	**0,001**	-7.5 (-45.2 - 25.5)	0,989
	Estradiol	57 (46 - 78)	-15.0 (-35.0 - 29.5)		-1 (-32 - 30)	
**estrone** *pg/mL*	Placebo	52 (37 - 67)	-19 (-32 - -11)	**0,001**	-1 (-16 - 13)	0,989
	Estradiol	55.0 (39.0 – 68.0)	3.0 (-10.5 - 20.0)		0.0 (-10.5 - 23.0)	
**FSH** *mIU/mL*	Placebo	5.95 (4.40 - 8.02)	-1.10 (-3.35 - -0.05)	**0,001**	0.00 (-1.15 - 1.40)	0,471
	Estradiol	6.60 (4.95 - 9.15)	-5.10 (-7.25 - -3.05)		-1.85 (-3.03 - 0.45)	
**LH** *mIU/mL*	Placebo	4.60 (3.55 - 5.10)	-4.00 (-4.88 - -2.88)	0,768	-0.700 (-1.450 - 0.725)	0,815
	Estradiol	4.90 (3.00 - 6.55)	-4.70 (-6.05 - -2.70)		-0.70 (-3.35 - 0.35)	
**progesterone** *ng/mL*	Placebo	0.4 (0.3 - 0.7)	-0.1 (-0.4 - 0.0)	0,989	0.0 (-0.2 - 0.2)	0,760
	Estradiol	0.4 (0.3 - 0.6)	-0.1 (-0.2 - 0.0)		0.1 (-0.2 - 0.6)	
**SHBG** *nmol/L*	Placebo	52 (30 - 63)	-8 (-16 - -2)	0,094	2 (-6 - 5)	0,760
	Estradiol	35.0 (29.5 - 54.0)	-2.0 (-6.5 - 8.5)		-1.0 (-5.0 - 1.5)	
**testosterone** *ng/dL*	Placebo	28.0 (17.0 - 32.2)	-4.0 (-10.8 - 0.0)	0,760	0.0 (-2.0 - 6.9)	0,760
	Estradiol	29.0 (23.5 - 35.5)	-4.0 (-8.5 - 1.0)		0.0 (-3.0 – 7.0)	

p values describe statistical significance of difference between estradiol and placebo group after intervention (p_I) and recovery (p_R). p values smaller than 0.05 in bold.
IQR – limits of the interquartile range (1st quartile - 3rd quartile).
FSH - Follicle-stimulating hormone; LH - Luteinizing hormone; SHBG - sex hormone-binding globulin

Supplementary Table 3. Relationship between a change in estradiol concentration and change in glycan abundance.

Glycan	Beta ± SE	p value
**GP1**	-0,079 ± 0,122	0,565
**GP2**	**-0,281 ± 0,088**	**0,008**
**GP3**	-0,256 ± 0,122	0,073
**GP4**	**-0,220 ± 0,080**	**0,021**
**GP5**	-0,208 ± 0,121	0,136
**GP6**	-0,019 ± 0,084	0,858
**GP7**	-0,228 ± 0,112	0,082
**GP8**	**-0,217 ± 0,090**	**0,039**
**GP9**	**-0,299 ± 0,084**	**0,007**
**GP10**	-0,094 ± 0,120	0,489
**GP11**	-0,19 0± 0,108	0,136
**GP12**	**0,282 ± 0,082**	**0,007**
**GP13**	**0,360 ± 0,115**	**0,009**
**GP14**	0,124 ± 0,081	0,169
**GP15**	**0,209 ± 0,085**	**0,037**
**GP16**	-0,027 ± 0,130	0,858
**GP17**	**0,400 ± 0,096**	**0,003**
**GP18**	**0,222 ± 0,078**	**0,017**
**GP19**	-0,208 ± 0,127	0,147
**GP20**	**0,430 ± 0,115**	**0,007**
**GP21**	0,138 ± 0,117	0,313
**GP22**	0,121 ± 0,132	0,437
**GP23**	0,180 ± 0,109	0,147
**GP24**	-0,124 ± 0,130	0,428
**B**	-0,014 ± 0,107	0,893
**CF**	**-0,295 ± 0,118**	**0,036**
**HM**	-0,208 ± 0,121	0,136
**G0**	**-0,195 ± 0,078**	**0,036**
**G1**	**-0,253 ± 0,083**	**0,011**
**G2**	0,133 ± 0,077	0,136
**G**	0,086 ± 0,099	0,452
**S0**	**-0,271 ± 0,081**	**0,007**
**S1**	**0,249 ± 0,077**	**0,008**
**S2**	0,255 ± 0,116	0,064
**S**	**0,270 ± 0,081**	**0,007**
**S/G**	**0,313 ± 0,090**	**0,007**

Beta = standardized coefficient; SE = standard error; GP = glycan peak in UPLC chromatogram; B = glycans with bisecting GlcNAc; CF = glycans with core fucosylation; HM = highly mannosylated glycans; G0 = agalactosylated glycans; G1 = monogalactosylated glycans; G2 = digalactosylated glycans; G = galactosylated glycans; S1 = monosialylated glycans; S2 = disialylated glycans; S = sialylated glycans; S/G = ratio of sialylation and galactosylation; B = glycans with bisecting GlcNAc

Supplementary Table 4. Changes in gene expression after transcriptional activation using dCas9-VPR or silencing, using dCas9-KRAB, of targeted loci. Reproducible significant results are shaded. Given p-values (p-val) are for Mann-Whitney test. Statistical significance: * $p<0.05$; ** $p<0.01$; *** $p<0.001$; ns = not significant.

		1st replicate			2nd replicate			pooled observations		
Targeted locus	Probed locus	ΔΔCt	fold change	p-val	ΔΔCt	fold change	p-val	ΔΔCt	fold change	p-val
RUNX1 / VPR	RUNX1	-1.61	3.054	0.002 (*)	-1.66	3.166	0.002 (*)	-1.64	3.110	0.000 (***)
RUNX1 / KRAB	RUNX1	0.18	0.883	0.589 (ns)	0.21	0.866	0.393 (ns)	0.19	0.875	0.378 (ns)
RUNX3 / VPR	RUNX3	-2.53	5.781	0.002 (*)	-2.46	5.510	0.002 (*)	-2.50	5.644	0.000 (***)
RUNX3 / KRAB	RUNX3	1.12	0.460	0.002 (*)	0.64	0.642	0.002 (*)	0.88	0.544	0.000 (***)
SPINK4 / VPR	SPINK4	-4.76	27.093	0.002 (*)	-4.34	20.221	0.002 (*)	-4.55	23.406	0.000 (***)
ELL2 / KRAB	ELL2	0.00	0.998	1.000 (ns)	0.65	0.636	0.002 (*)	0.33	0.796	0.319 (ns)
RUNX3 / VPR	B4GALT1	-0.04	1.025	0.818 (ns)	/	/	/	-0.04	1.025	0.818 (ns)
SPINK4 / VPR	B4GALT1	0.31	0.804	0.008 (**)	0.18	0.880	0.180 (ns)	0.25	0.841	0.160 (ns)
SPINK4 / VPR	ST6GAL1	-0.09	1.065	0.589 (ns)	/	/	/	-0.09	1.065	0.589 (ns)

Supplementary Table 5. Raw experimental data for glycan traits in gene activation and repression by dCas9-VPR and -KRAB fusions targeted using specific gRNA to individual loci. Glycan traits represent sums of absolute percentages of relevant glycan peaks. These data were used to construct Figure 3. Results for loci that did not result in a significant change in glycans (not shown in Figure 3) are also given. Comparison was made between "gRNA" and "NT" (non-targeting, negative control) sample sets. Glycan traits - **G0** (% of agalactosylated glycans) is: GP1, GP2, GP3, GP4, GP6; **G** (% of galactosylated glycans) is: GP7, GP8, GP9, GP10, GP11, GP12, GP13, GP14, GP15; **G1** (% of monogalactosylated glycans) is: GP7, GP8, GP9, GP10, GP11; **G2** (% of digalactosylated glycans) is: GP12, GP13, GP14, GP15. Note that **G** = **G1** + **G2**.

Locus	Trait	Values / %	dCas9 fusion	Experiment
B4GALT1	G0	77.59, 81.17, 80.40, 81.61, 75.62, 78.60	KRAB	1/1
	G	15.15, 12.12, 11.91, 11.80, 13.53, 12.48		
	G1	11.92, 10.10, 9.72, 10.46, 10.45, 10.69		
	G2	3.24, 2.02, 2.19, 1.34, 3.07, 1.80		
	G0	60.75, 55.43, 61.31, 39.28, 61.01, 65.56	NT	
	G	28.13, 26.55, 25.15, 33.28, 25.32, 28.27		
	G1	21.20, 19.78, 20.19, 22.03, 19.54, 23.48		
	G2	6.93, 6.77, 4.95, 11.25, 5.78, 4.79		
RUNX3	G0	68.70, 67.57, 68.22, 66.13, 63.18, 66.59	VPR	1/2
	G	24.39 25.70, 25.27, 25.40, 27.17, 26.13		
	G1	21.44, 21.88, 21.46, 21.66, 22.85, 21.78		
	G2	2.95, 3.82, 3.81, 3.74, 4.32, 4.35		
	G0	62.33, 62.21, 63.46, 55.32 53.90	NT	
	G	29.79, 31.66, 30.84, 34.42, 37.49		
	G1	25.39, 26.62, 26.25, 27.12, 30.16		
	G2	4.41, 5.04, 4.59, 7.30, 7.33		
	G0	69.75, 68.47, 70.26, 71.71, 66.51, 72.56	VPR	2/2
	G	23.90, 24.25, 21.79, 22.02, 20.65, 21.82		
	G1	20.47, 20.60, 18.87, 19.33, 16.87, 18.21		
	G2	3.44, 3.65, 2.92, 2.68, 3.78, 3.61		
	G0	64.98, 66.70, 60.59, 67.82, 69.11, 68.36	NT	
	G	27.65, 25.42, 29.27, 26.40, 25.84, 27.79		
	G1	23.52, 22.20, 23.55, 22.89, 22.57, 24.05		
	G2	4.13, 3.22, 5.72, 3.50, 3.27, 3.74		
SPINK4	G0	57.03, 55.18, 66.55, 56.21, 54.76, 59.19	VPR	1/2
	G	29.21, 25.22, 26.24, 30.50, 27.84, 25.18		
	G1	21.98, 20.06, 22.18, 22.82, 21.21, 20.16		
	G2	7.22, 5.16, 4.06, 7.69, 6.63, 5.02		
	G0	62.33, 62.21, 63.46, 55.32 53.90	NT	
	G	29.79, 31.66, 30.84, 34.42, 37.49		
	G1	25.39, 26.62, 26.25, 27.12, 30.16		
	G2	4.41, 5.04, 4.59, 7.30, 7.33		

	G0	71.83, 69.31, 66.49, 68.93, 72.57, 66.59	VPR	2/2
	G	22.58, 25.59, 24.66, 19.52, 21.71, 26.70	VPR	2/2
	G1	19.61, 22.18, 18.92, 16.75, 18.83, 22.26	VPR	2/2
	G2	2.97, 3.40, 5.75, 2.77, 2.88, 4.44	VPR	2/2
	G0	64.98, 66.70, 60.59, 67.82, 69.11, 68.36	NT	2/2
	G	27.65, 25.42, 29.27, 26.40, 25.84, 27.79	NT	2/2
	G1	23.52, 22.20, 23.55, 22.89, 22.57, 24.05	NT	2/2
	G2	4.13, 3.22, 5.72, 3.50, 3.27, 3.74	NT	2/2
Locus	Trait	Values / %	dCas9 fusion	Experiment
RUNX1	G0	60.89, 59.07, 64.33, 56.27, 52.58, 58.52	VPR	1/1
RUNX1	G	30.30, 34.70, 28.00, 35.02, 37.63, 32.57	VPR	1/1
RUNX1	G1	24.30, 28.29, 23.69, 29.70, 29.84, 25.99	VPR	1/1
RUNX1	G2	6.00, 6.41, 4.31, 5.32, 7.79, 6.58	VPR	1/1
RUNX1	G0	62.33, 62.21, 63.46, 55.32 53.90	NT	1/1
RUNX1	G	29.79, 31.66, 30.84, 34.42, 37.49	NT	1/1
RUNX1	G1	25.39, 26.62, 26.25, 27.12, 30.16	NT	1/1
RUNX1	G2	4.41, 5.04, 4.59, 7.30, 7.33	NT	1/1
RUNX1	G0	68.61, 66.89, 61.98, 66.16, 67.10, 67.27	VPR	2/2
RUNX1	G	24.43, 26.70, 25.95, 28.35, 26.41, 23.26	VPR	2/2
RUNX1	G1	21.19, 23.00, 21.65, 24.16, 22.27, 19.83	VPR	2/2
RUNX1	G2	3.23, 3.71, 4.30, 4.19, 4.14, 3.43	VPR	2/2
RUNX1	G0	64.98, 66.70, 60.59, 67.82, 69.11, 68.36	NT	2/2
RUNX1	G	27.65, 25.42, 29.27, 26.40, 25.84, 27.79	NT	2/2
RUNX1	G1	23.52, 22.20, 23.55, 22.89, 22.57, 24.05	NT	2/2
RUNX1	G2	4.13, 3.22, 5.72, 3.50, 3.27, 3.74	NT	2/2
RUNX1	G0	44.83, 54.34, 52.65, 53.24, 49.80, 43.74	KRAB	1/1
RUNX1	G	36.33, 31.23, 29.88, 35.00, 32.47, 32.39	KRAB	1/1
RUNX1	G1	25.91, 25.13, 24.17, 29.13, 25.53, 22.28	KRAB	1/1
RUNX1	G2	10.41, 6.11, 5.71, 5.87, 6.93, 10.11	KRAB	1/1
RUNX1	G0	60.75, 55.43, 61.31, 39.28, 61.01, 65.56	NT	1/1
RUNX1	G	28.13, 26.55, 25.15, 33.28, 25.32, 28.27	NT	1/1
RUNX1	G1	21.20, 19.78, 20.19, 22.03, 19.54, 23.48	NT	1/1
RUNX1	G2	6.93, 6.77, 4.95, 11.25, 5.78, 4.79	NT	1/1
RUNX1	G0	77.22, 78.08, 75.64, 77.24, 69.33, 74.23	KRAB	2/2
RUNX1	G	17.47, 17.40, 16.44, 17.42, 25.18, 18.32	KRAB	2/2
RUNX1	G1	15.40, 15.23, 14.77, 15.46, 20.53, 15.09	KRAB	2/2
RUNX1	G2	2.07, 2.18, 1.67, 1.96, 4.65, 3.23	KRAB	2/2
RUNX1	G0	75.84, 76.98, 78.08, 72.77, 66.06, 74.83	NT	2/2
RUNX1	G	17.72, 18.51, 18.20, 21.38, 19.50, 20.05	NT	2/2
RUNX1	G1	15.15, 16.22, 16.22, 17.66, 15.92, 17.43	NT	2/2
RUNX1	G2	2.57, 2.28, 1.98, 3.72, 3.58, 2.62	NT	2/2
RUNX3	G0	50.38, 51.25, 50.47, 54.80, 59.60, 48.60	KRAB	1/2
RUNX3	G	35.13, 32.66, 32.38, 34.67, 34.88, 33.54	KRAB	1/2
RUNX3	G1	27.88, 25.10, 24.96, 28.04, 29.14, 26.58	KRAB	1/2
RUNX3	G2	7.24, 7.57, 7.41, 6.63, 5.74, 6.96	KRAB	1/2

	G0	60.75, 55.43, 61.31, 39.28, 61.01, 65.56	NT	
	G	28.13, 26.55, 25.15, 33.28, 25.32, 28.27		
	G1	21.20, 19.78, 20.19, 22.03, 19.54, 23.48		
	G2	6.93, 6.77, 4.95, 11.25, 5.78, 4.79		
	G0	77.46, 77.67, 73.85, 71.52, 77.34, 71.15	KRAB	2/2
	G	17.94, 17.85, 19.23, 17.24, 18.60, 20.85		
	G1	15.61, 15.76, 16.09, 14.44, 16.48, 18.32		
	G2	2.33, 2.08, 3.14, 2.79, 2.13, 2.53		
	G0	75.84, 76.98, 78.08, 72.77, 66.06, 74.83	NT	
	G	17.72, 18.51, 18.20, 21.38, 19.50, 20.05		
	G1	15.15, 16.22, 16.22, 17.66, 15.92, 17.43		
	G2	2.57, 2.28, 1.98, 3.72, 3.58, 2.62		
ELL2	G0	54.80, 44.56, 46.96, 47.55, 42.07, 41.05	KRAB	1/2
	G	33.26, 30.64, 29.09, 34.12, 38.70, 41.56		
	G1	27.24, 20.08, 18.28, 26.50, 27.69, 26.79		
	G2	6.02, 10.56, 10.81, 7.62, 11.01, 14.77		
	G0	60.75, 55.43, 61.31, 39.28, 61.01, 65.56	NT	
	G	28.13, 26.55, 25.15, 33.28, 25.32, 28.27		
	G1	21.20, 19.78, 20.19, 22.03, 19.54, 23.48		
	G2	6.93, 6.77, 4.95, 11.25, 5.78, 4.79		
	G0	71.54, 78.77, 75.81, 71.19, 75.26, 73.37	KRAB	2/2
	G	17.61, 16.81, 19.39, 17.11, 16.98, 20.52		
	G1	14.19, 15.13, 16.45, 14.04, 14.32, 16.20		
	G2	3.42, 1.68, 2.94, 3.07, 2.66, 4.32		
	G0	75.84, 76.98, 78.08, 72.77, 66.06, 74.83	NT	
	G	17.72, 18.51, 18.20, 21.38, 19.50, 20.05		
	G1	15.15, 16.22, 16.22, 17.66, 15.92, 17.43		
	G2	2.57, 2.28, 1.98, 3.72, 3.58, 2.62		

Supplementary Table 6. Sequences of gRNA molecules used in each experiment.

sgRNA molecule	Target site sequence (5'→3')	Experiment
RUNX1_A-sg01	GCCTGCGTGTGGCTGCGGAA	Overexpression with dSaCas9-VPR
RUNX1_A-sg02	GCCCCTATGCAAACGAGCTGA	
RUNX1_A-sg03	GAGTAGACTTTGCAAGAGGG	
RUNX3_A-sg01	GTGGTGGGTACGAGGGCGGCC	
RUNX3_A-sg02	GTGGCACTGGGGCCTCCTCCG	
RUNX3_A-sg03	GGGCCTGCCGGAGGCCGCCCA	
SPINK4_A-sg01	GCACCTAGAGCAAGCCCTTT	
SPINK4_A-sg02	GGCCCTAGATAAGATAAGATT	
SPINK4_A-sg03	GCCATGGGGCTGGCAGGTTTC	
B4GALT1_A-sg01	GCGACTAGGGGAGGGCCCGGA	
RUNX1_G-sg01	GGGCTGCGTACAGTAGCGCG	Silencing with dSpCas9-KRAB
RUNX1_G-sg02	GAGCCGAGTAGACTTTGCAAG	
RUNX1_G-sg03	GGCAGCGTGGTGCCCTGGCT	
RUNX3_G-sg01	GCACCTCGGTGGCGTTCGCG	
RUNX3_G-sg02	GCGCGGGGTTAGTACCCCCG	
RUNX3_G-sg03	GGACCGGGAATCCAAATTCT	
ELL2_G-sg01	GTCCGGCTCTAGCCTCCACTG	
ELL2_G-sg02	GCTATGGGCTGTCGTGCGGA	
ELL2_G-sg03	GCCGCCATCTTAAACTCCCC	
B4GALT1_G-sg01	GTAGGGACGCGCCTGGCATCG	
NT-sgRNA	GTAGGCGCGCCGCTCTCTAC	Non-targeting control

Supplementary Table 7. Details of plasmid constructs used for modular Golden Gate cloning method (Josipović et al. 2019).

Plasmid	Description	Resistance	Ends	Restriction	Selection
pSgM1-A	SaCas9 sgRNA scaffold, U6 promoter, position 1	Spectinomycin	B-A	Esp3I; BpiI (sgRNA)	red-white for gRNA
pSgM2-A	SaCas9 sgRNA scaffold, U6 promoter, position 2	Spectinomycin	A-I	Esp3I; BpiI (sgRNA)	red-white for gRNA
pSgM3-A	SaCas9 sgRNA scaffold, U6 promoter, position 3	Spectinomycin	I-II	Esp3I; BpiI (sgRNA)	red-white for gRNA
pSgM1-G	SpCas9 sgRNA scaffold, U6 promoter, position 1	Spectinomycin	B-A	Esp3I; BpiI (sgRNA)	red-white for gRNA
pSgM2-G	SpCas9 sgRNA scaffold, U6 promoter, position 2	Spectinomycin	A-I	Esp3I; BpiI (sgRNA)	red-white for gRNA
pSgM3-G	SpCas9 sgRNA scaffold, U6 promoter, position 3	Spectinomycin	I-II	Esp3I; BpiI (sgRNA)	red-white for gRNA
pSgx1	Single sgRNA module, accepts 1 sgRNA	Kanamycin	B-A; B-A (out)	BsaI; Esp3I (inverted)	red-white for modules
pSgx3	Multiple sgRNA module, accepts 3 sgRNA	Kanamycin	B-A; B-II (out)	BsaI; Esp3I (inverted)	red-white for modules
pBackBone-BZ	Backbone for assembly	Ampicillin	B-Z (out)	BsaI (inverted)	blue-white
tNS-pPro-CBh	Cbh promoter	Kanamycin	A-I	BsaI	none
pHC-1-f3 (module for heavy chain)	Heavy chain of IgG	Kanamycin	I-III	BsaI	none
pLC-Int-T2A (module for light chain)	Light chain of IgG	Kanamycin	III-IV	BsaI	none
tNS-pTer-H	Bgh transcriptional terminator	Kanamycin	IV-Z	BsaI	none

Supplementary Table 8. Primer sequences.

Primer	Sequence (5'→3')	Use
RUNX3 Fw	CAGATCCTGGCCGTCTCATC	Amplification of *RUNX3* transcript with SYBR QPCR
RUNX3 Rev	GGCTTATCCTGTGGGCCAAT	
HPRT1 Fw	CCTGGCGTCGTGATTAGTGAT	Amplification of *HPRT1* transcript with SYBR QPCR
HPRT1 Rev	AGACGTTCAGTCCTGTCCATAA	
